# Bridging the Gap: Evaluating Global Health Training Needs in Pediatric Residency and Fellowship: A Program‑Wide Analysis

**DOI:** 10.5334/aogh.4734

**Published:** 2025-08-28

**Authors:** Allyson Rose, Riad Rahhal, Dina Al-Zubeidi

**Affiliations:** 1 Carver College of Medicine, Global Health Distinction Track, University of Iowa, USA; 2 Clinical Professor of Pediatrics, Section Chief, Pediatric Gastroenterology, Hepatology and Nutrition, Department of Pediatrics, University of Illinois College of Medicine‑Peoria, USA; 3 Clinical Associate Professor of Pediatrics, Associate Fellowship Program Director, Pediatric Gastroenterology Hepatology Pancreatology and Nutrition, Stead Family Department of Pediatrics, Carver College of Medicine, University of Iowa, USA

**Keywords:** global health education, pediatric global health, pediatric medical education

## Abstract

*Background:* Interest in global health (GH) among pediatric trainees has grown, yet structured GH training remains limited in many programs.

*Objective:* This study evaluates the GH interests, perceived barriers, and curriculum needs of pediatric residents and fellows in a Midwestern training program.

*Methods:* A 23‑question anonymous survey was administered to pediatric trainees between September 2023 and September 2024. The survey assessed demographics, GH interests, barriers to participation, and curriculum gaps. Respondents were categorized by American medical graduates (AMG) vs. international medical graduates (IMG) and junior (PGY1‑2) vs. senior (PGY3‑7) trainees. Statistical analysis was conducted using SAS 9.4.

*Results:* The survey response rate was 60%. Even though only 6% prioritized GH training in program selection, 56% expressed interest in GH electives. Time constraints (34%) and family responsibilities (18%) were primary barriers. IMGs (90%) were more likely than AMGs (65%) to express GH career interest. Communication skills development was identified as the highest educational need overall for all trainees, with junior trainees prioritizing it (59% vs. 32% seniors, p = 0.056) and AMGs vs. IMGs, 53% and 22% of the time (p = 0.15).

*Conclusions:* Our study sheds light on differences in GH interest and trainee characteristics throughout pediatric training from residency through fellowship, considering AMG vs. IMG as a factor. Further, we were able to note how the desire for increased curriculum time in some of the ACGME’s core competencies changes throughout the advanced training years, which helps guide further curriculum development. In addition, we uncovered that, despite our program’s lack of GH focus, interest in GH remains high, and we call to action for further GH‑focused curriculum exposure in all pediatric training programs, regardless of size and location.

## Background

Global health (GH) is an area of study, research, and practice that focuses on worldwide improvement of health, reduction of disparities, and protection against global threats that disregard national borders. Interest in GH has steadily increased among researchers and medical care providers over the last several decades due to events such as global epidemics and human migration [1]. Due to advancements in technology and transportation, we see that there is no such thing as an entirely geographically isolated health issue. Diseases and outbreaks are not limited by borders or even oceans. For this reason, the Lancet Commission Report on medical education for the 21st century urged that, “all health professionals in all countries should be educated to mobilize knowledge and to engage in critical reasoning and ethical conduct so that they are competent to participate in patient and population‑centered health systems as members of locally responsive and globally connected teams” [2].

Greater than 90% of pediatric deaths occur in low‑ and middle‑income countries (LMICs) where there is a severe shortage of pediatricians [3, 4]. This tragic reality, along with increased interest from trainees, has translated into academic medical programs increasing their formal training in matters concerning GH. According to one survey in 2014, 58% of pediatric residency programs offer international field experience [5]. Further, approximately one fourth of pediatric residencies offer a dedicated GH track [6]. Many medical students even state that opportunities to train in GH were an essential factor in selecting a training program [7].

Our postgraduate training program does not offer a specific GH track for pediatric trainees. Trainees can, however, participate in GH trips if they can arrange it. In addition, pediatric GH education is incorporated into the curriculum during one full education day for residents once every few years, but not for fellows. Pediatric training programs aim to prepare future pediatricians to provide appropriate care to children who come from many diverse types of cultural backgrounds or resource settings. Educating residents and fellows on GH issues provides the opportunity to aid in the development of skills that may be utilized to help children both in the United States (US) and abroad. [4]. Studies show that training in GH improves expertise on immigrant health, improves care for adopted children, and is directly applicable to providing care in low‑resource settings [6, 8, 9]. In addition, GH training improves medical knowledge, diagnostic skills, enhances cultural humility, gives a broader understanding of resource utilization, and increases awareness of social determinants of health [5]. In these ways, the skills acquired from GH‑focused training increase the competence of domestic physicians, especially when caring for immigrant or vulnerable populations within the US [6]. Experiences with GH have been shown to increase the participants’ interest in entering primary care fields and increase the likelihood of those individuals practicing in underserved settings, whether it be locally or abroad [10, 11].

This study employed a survey approach aimed at gaining insight as to how local or international rotations should be structured, measuring trainee baseline interest in GH, identifying barriers toward participation in GH efforts, gathering information about prior GH, and identifying trainee characteristics that predict future involvement in GH. Additionally, the survey is a form of needs assessment that aims to aid in further GH curriculum design for the pediatric residency/fellowship trainees. This survey was employed on a program that does not have a formal GH focus and, hence, it provides insight that may be particularly relevant for similar programs who may not have perceived the importance of GH and may need to reconsider more GH integration in their training curriculum.

## Methods

A survey was developed and underwent IRB review and approval at the University of Iowa. The survey was created on Microsoft Forms software and distributed to all pediatric trainees in attendance at different educational sessions from September 2023 to September 2024. Informed consent was provided with the option to opt out. The survey included up to 23 questions in total with a combination of multiple‑choice questions, five‑point Likert scale questions, and free text questions. The survey took less than 3 minutes to complete. The number of survey questions presented was dependent on how respondents answered specific questions, as a branching algorithm was utilized. To maintain the anonymity of respondents, names or email addresses were not recorded. There was no compensation provided to fill out.

The first section collected demographic information including age, gender, ethnicity, marital status, parental status, location of medical school, and location of primary residence while growing up, and a map was provided with this question to identify regions of the US separated by state to maintain consistency among respondents. The second section inquired about prior participation in GH efforts and how it impacted the respondent’s interest in the field. The third section sought to elucidate the respondent’s current interest in GH electives, future interest in a career including GH, the role that the presence of a GH program played in residency/fellowship selection, and barriers to participation. Finally, the last set of questions was about curriculum needs among trainees based on Accreditation Council for Graduate Medical Education (ACGME) competencies.

Responses were compiled and separated into categories for analysis based on American (AMG) vs. international (IMG) medical school attendance and postgraduate year (PGY) training level as junior (first‑ and second‑year residents) vs. senior (third‑year residents and fellows) trainee status. Responses on the likelihood of pursuing GH following medical training were converted to a binary scale, as any indication of pursuing any GH in any manner was categorized as a positive response. Comparisons were made between AMG vs. IMG and junior vs. senior trainees on questions regarding the likelihood of pursuing GH, identification of time or family as a primary barrier to pursuit of GH, and whether they identified interpersonal and communication skills as their primary gap in the current GH curriculum.

Continuous data were reported as means with ranges. Categorical variables were described as percentages and compared by a chi‑square test or Fisher’s exact test as appropriate. A probability value < 0.05 was considered significant. Statistical analysis was performed using SAS 9.4 (SAS Institute, Cary, NC).

## Results

### Demographics

A total of 38 pediatric residents (of 50 total residents) and 12 pediatric fellows (of 34 total fellows) completed the survey for a survey response number of 50. The response rate was 60%. The mean age of respondents was 29.3 years with a range of 23–36 years. Approximately 76% of all respondents identified as female. The majority (68%) of participants self‑identified as white. Junior residents (PGY1‑2) made up 54% of respondents (37) with senior residents (PGY3‑7) comprising the other half. Fifty percent of respondents were married. Most participants (80%) were not a parent or guardian to any children under the age of 17. Twenty percent of respondents were graduates of international medical schools with degrees from four different continents being represented. When asked about where they lived while growing up, 70% indicated the Midwest within the US and 10% primarily grew up abroad (Table 1).

**Table 1:** Demographics.

**SOCIODEMOGRAPHIC DEMOGRAPHIC CHARACTERISTICS OF RESPONDENTS**			
CHARACTERISTICS	ALL	JUNIOR TRAINEES (PGY1‑2)	SENIOR TRAINEES (PGY3‑7)
Number, n	50	27	23
Sex, n	38	22	14
Female	38	22	14
Male	11	3	8
Other	1	1	0
Ethnicity			
Caucasian	34	22	12
Middle Eastern	5	1	4
Asian	5	2	3
Hispanic/Latino	2	1	1
Indian	1	0	1
Black/African American	1	1	0
From Multiple Races	2	0	2
Marital Status			
Married	25	13	12
Never Married	25	14	11
Children			
0	40	22	18
1–2	10	5	5
Location of Medical School			
United States (AMG)	40	24	16
International (IMG)	10	3	7

### Participation in GH

Of the 50 respondents, 72% had never participated in any GH prior to entering pediatric training. Of the 14 respondents (28%) who had GH experience prior to residency, only two trainees had participated in GH work while in training (14%). Of the prior GH exposures, 43% participated in both international and domestic electives, 21% domestic, and 28% international. Seventy‑nine percent of respondents indicated that their GH experience has increased their desire to pursue a career in GH. No respondents indicated that their experience dissuaded them from a career in GH.

Among those with prior GH experience, approximately 92% indicated plans to engage in GH efforts during their career, whether it be through long‑term opportunities (79%) or short‑term opportunities (14%). Notably, those without any prior GH experience did not pursue any GH efforts during training despite interest.

### Current GH interest

Among all 50 respondents, 56% indicated they would (very or somewhat likely) participate in a GH elective if offered during their training program. Although this interest exists, only 6% of respondents indicated that they highly prioritized the availability of GH training when choosing a residency or fellowship program. Further, 42% of respondents did not prioritize the availability of GH training at all when choosing their training program.

### Career goals

When analyzing the duration of commitment by which respondents intend to participate in GH efforts, 32% indicated they were very likely to seek short‑term opportunities only. Ten percent indicated they have definite plans or are very likely to work in long‑term positions following graduation. Approximately 28% of respondents indicated they were somewhat likely to seek a long‑term position. Seventy percent of trainees want to participate in GH work within the US or internationally during their career. However, only 4% (2 trainees) of our cohort noted participation in GH throughout training and 63% of those who noted they intended to participate in GH in their career had no prior experience in GH work or studies.

**Figure 1:**
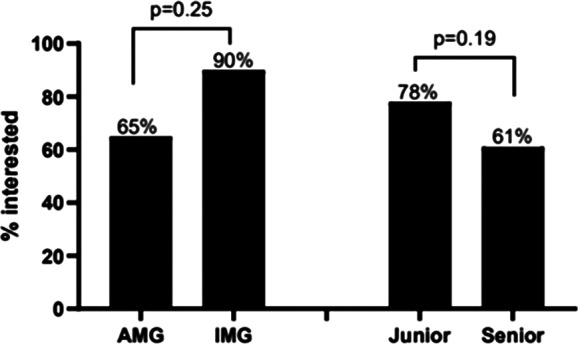
Interest to pursue global health efforts post training.

A difference was noted when comparing AMG vs. IMG regarding interest in GH at 65% and 90% respectively, but this did not reach statistical significance (p = 0.25). Junior trainees (PGY1‑2) had a slightly higher interest in a career involving GH (78%) vs. senior trainees (PGY 3‑7) at 61% (p = 0.19); (Figure 1).

### Program‑specific curriculum

Sixty percent reported their experiences in residency or fellowship have been neutral in preparing them to address the concerns of pediatric GH, while 28% noted they felt they had poor or very poor preparation.

### Barriers to pursuit of GH

When looking at the barriers to participation, 17 respondents (34%) identified time while 9 (18%) identified family responsibilities as their primary barriers to participating in GH. Time constraints were identified as the main barrier by 33% and 40% of AMGs and IMGs responsively (p = 0.72).

Family reasons were identified as the main barrier by 23% and 0% among AMGs and IMGs responsively (p = 0.17). Junior and senior trainees noted time barrier to participation similarly at 33% and 35%, respectively (p = 0.91), and family barrier at 22% and 13%, respectively (p = 0.48).

Regarding the barrier of family responsibilities, we see that all those who identified this cause as their barrier were AMGs. This indicates that while the location of medical school attendance may not influence the fact that a lack of time presents a barrier to participating in GH, there may be differences in family lifestyle between IMGs and AMGs such that this barrier exists more prominently in the AMG group. One observation in our cohort was that junior trainees were more likely to identify family responsibilities as their primary barrier to pursuit of GH than senior trainees were. Therefore, we may conclude that junior pediatric trainees are in greater need of resources for support in the maintenance of the family structure so that they may pursue their educational goals, including GH.

**Figure 2:**
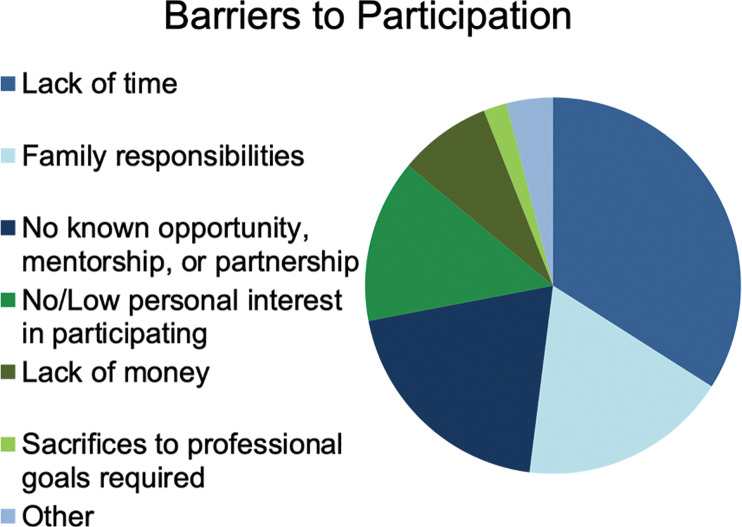
Barriers to participation.

**Figure 3:**
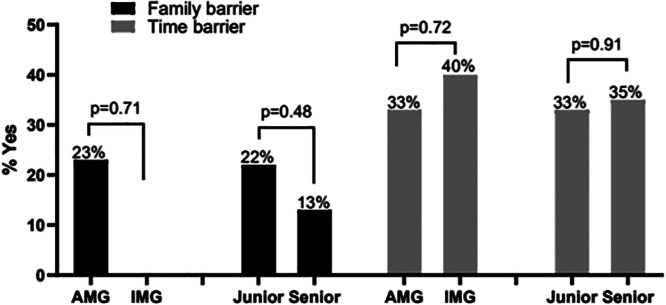
Family and Time as primary barriers to pursue global health efforts.

In terms of funding, only 38% of trainees were aware of any financial support available to help cover costs of expenses associated with participating in GH experiences. However, just 8% stated that their primary barrier to participating in a GH elective was financial (Figures 2 and 3).

### Curriculum needs among trainees

Regarding the preference to see more educational time dedicated to ACGME‑based competencies, a top priority (44%) was dedicating more time to communicating effectively with patients, families, and the public, coming from a broad range of cultural and socioeconomic backgrounds. Others prioritized developing medical knowledge on the presentation, diagnosis, management, and prevention of global infections (26%), performing essential components of a history and physical examination for children with immigrant or refugee status (16%), reflecting on personal bias that may impact clinical decision‑making when caring for diverse and vulnerable populations (12%), or a combination of the choices.

When comparing responses from AMGs vs. IMGs, communication skills were identified as a gap in training 53% and 22% of the time (p = 0.15).

Therefore, although these results were not found to be statistically significant, we suspect there is a difference between the curriculum needs of IMGs and AMGs regarding communication skills.

Juniors and seniors prioritized communication skills 59% and 32% of the time (p = 0.056).

**Figure 4:**
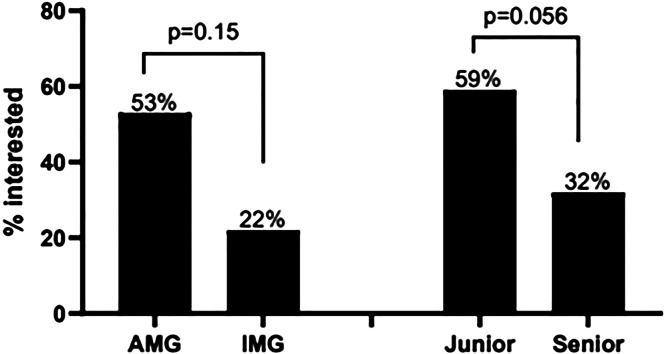
Curriculum needs among trainees: need for communication skills development in GH: based on Accreditation Council for Graduate Medical Education (ACGME) competencies.

Seniority impacted what trainees found to be their biggest curriculum gap. Seventy percent of those who wanted more exposure to interprofessional and communication skills were junior trainees. Senior trainees were more likely to identify a different gap in the GH curriculum. As this result approached statistical significance (p = 0.056), we noted that our cohort desires earlier exposure to the ACGME core competency of interprofessional and communication skills (Figure 4).

## Discussion

Prior research aiming to identify the qualities of medical trainees associated with successful pursuit of GH, barriers to participation, and GH curriculum needs is mostly based on surveys of program directors, residents, or fellows following graduation of a GH track, or of medical students [6, 12]. Our survey adds to the field by allowing the comparison of responses throughout pediatric training instead of before or after training entirely. Further, we stratified our results to identify differences in GH interest and experience between AMGs and IMGs and seniority level. Of note, we elucidate responses from trainees at a program without a specific GH focus/track.

Our results uncover that pediatric trainees are interested in GH work irrespective of whether they had GH exposure prior to training, or if they had not prioritized that during program selection, even still if they are currently training in a program without a GH focus. Particularly those trainees with prior exposure to GH remained interested, and a small number even pursued GH during their training despite the lack of GH focus at our institution. This aligns with previously reported results that prior pursuit of GH is a positive predictor of future engagement in GH efforts [13]. These results support the increased need for resources and support for educating pediatric residents and fellows on GH issues, specifically at smaller programs or institutions without a GH focus, as pediatricians are likely to pursue GH work at some point in their career.

A higher percentage of IMGs reported the likelihood of pursuing GH following training than AMGs, indicating attending medical school outside of the US may be predictive of increased desire to engage in GH. Additionally, we see that early in training, respondents were more likely to express a desire to engage in GH in their career than they were later in training. This result suggests that throughout pediatric medical training, there may be events or barriers that are created that reduce interest in engaging in GH work.

With lack of time identified as a major barrier, there is a need for dedicated time set aside in training programs in the pursuit of GH electives.

This report was collected from medium‑sized Midwestern pediatric residency/ fellowship programs. Most participants were female (76%), white (68%), and unmarried (50%). Most participants grew up in the Midwest (70%), with a small representation from medical schools outside of the US (10%). Therefore, we anticipate these results are representative of programs of comparable size or demographics. As this report was only collected from one center, the study is limited in the ability to generalize to all pediatric trainees’ experiences with GH. Further, the small sample size may contribute to why some trends were noted, but the results were not statistically significant.

## Conclusions

Most literature regarding the qualities and characteristics of pediatric trainees that predict pursuit of GH or evaluation of their desired learning outcomes have focused on graduates of a GH training program or medical students prior to participation in residency or fellowship. Our study sheds light on differences in GH interest and trainee characteristics throughout pediatric training from residency through fellowship and considers AMG vs. IMG as a factor. Further, we were able to note how specific curriculum needs in some of the ACGME’s core competencies change throughout the advanced training years. We have also uncovered that, despite the lack of robust GH opportunities in our program, the interest in pursuit of GH remains high and so is the likelihood of future GH engagement in our cohort of trainees and, hence, the authors eagerly call to action to similar training programs without a GH focus to integrate GH in training to allow for ethical and sustainable GH efforts once their trainees graduate or even if they engage in efforts during training.
